# Urethral deviation may be a potential pathogenic factor in female stress urinary incontinence: a cross-sectional study

**DOI:** 10.1097/JS9.0000000000003696

**Published:** 2025-10-16

**Authors:** Pan Hu, Xun Yang, Yajun Song, Jiang Zhao, Chunmei Xiao, Chunyan Zhong, Lubin Liu

**Affiliations:** a Department of Obstetrics and Gynecology, Women and Children’s Hospital of Chongqing Medical University, Chongqing, China; b Changchun Institute of Optics, Fine Mechanics and Physics, Chinese Academy of Sciences, Changchun, China; c Department of Urology, The Second Hospital Affiliated of the Army Medical University, Chongqing, China; d Department of Ultrasound, Women and Children’s Hospital of Chongqing Medical University, Chongqing, China

**Keywords:** finite element model, pelvic floor, stress urinary incontinence, urodynamics, women’s health

## Abstract

**Background:**

Stress urinary incontinence (SUI) is one of the top five chronic diseases globally. The traditional theory of SUI does not adequately explain certain subgroups of patients. This study aims to investigate the risk factors and potential mechanisms underlying female SUI.

**Materials and methods:**

A dual tertiary-center, cross-sectional study was conducted. A finite element model (FEM) was developed using data from a female volunteer. A total of 42 rats were utilized as animal models. Female participants presenting with lower urinary tract symptoms were recruited and categorized into four groups based on their SUI and Levator ani avulsion (LAA) status: SUI+/LAA+, SUI+/LAA−, SUI−/LAA+, and SUI−/LAA−. Data were collected from the FEM, animal models, and clinical participants.

**Results:**

The FEM demonstrated that in simulations of unilateral LAA, the ipsilateral urethra exhibited deviation and displacement toward the site of avulsion, accompanied by distortion. Rats with LAA showed a significantly higher incidence of SUI (*P* = 0.031), particularly those with unilateral LAA (*P* = 0.011). A total of 1629 women were ultimately included in the study. Statistical significance was observed specifically in patients with unilateral LAA (odds ratio = 1.87 [95% confidence interval, 1.389–2.481], *P* < 0.001). Measurements of the levator-urethral gap indicated that the closer the avulsion site was to the urethral opening, the higher the likelihood of SUI occurrence, which aligns with the urethral deviation patterns observed in the FEM analysis.

**Conclusion:**

Unilateral LAA is a significant risk factor for SUI. The urethral deviation induced by unilateral LAA may represent an additional etiological mechanism of SUI, beyond the traditional fascial hammock theory.

## Introduction

Stress urinary incontinence (SUI) is among the five most prevalent chronic conditions worldwide^[1]^. Although fewer than 40% of affected women seek medical care, epidemiological surveys indicate that the prevalence of SUI among adult women reaches up to 46%^[2,3]^. The prevalence increases with age, peaking at 50% among women aged 40 years and older^[4]^.

The classical hammock theory posits that SUI is associated with urethral hypermobility^[5]^. While this theory explains the majority of SUI mechanisms, it fails to account for certain atypical presentations, such as cases in patients without anterior vaginal wall prolapse – where urethral descent is absent yet SUI persists – or individuals who have undergone pelvic floor reconstruction surgery, which may reduce urethral mobility but still result in a significant incidence of postoperative *de novo* SUI. As previously demonstrated^[6,7]^, unilateral levator ani avulsion (LAA) may be a risk factor for SUI. Moreover, patients with LAA have been shown to exhibit a higher incidence of *de novo* SUI after pelvic floor reconstruction surgery, despite having normal anatomical structures and no evidence of urethral hypermobility^[7]^. Therefore, we hypothesize that alterations following LAA play a significant role in the development of SUI, thereby complementing the classical hammock theory.

In the present study, we employ medical simulation technology to investigate pelvic floor support structures, simulate levator ani muscle injuries using computer-based finite element modeling, and apply the Bernoulli fluid mechanics equation in conjunction with SUI animal models to elucidate the relationship between levator ani muscle injury and the development of SUI. Furthermore, population-based surveys are conducted to validate these findings, aiming to provide robust empirical support for a more comprehensive understanding of SUI etiology.

## Methods

### Part I: establishment of the female pelvic finite element model

A healthy female volunteer (48 years old, body mass index [BMI]: 21.47 kg/m^2^) with no history of pelvic surgery and no symptoms of pelvic floor dysfunction was recruited. Magnetic resonance imaging (MRI) images were reconstructed using the interactive medical image processing software Mimics 21.0. Each 3 dimentional (3D) geometric model was exported in STL format and imported into Geomagic Wrap for surface refinement and optimization.

The complete 3D geometric model was then imported into the finite element pre-processing software Hypermesh v.2021 to construct the finite element model (FEM). To simulate structural deformation under abdominal pressure, bones and muscles were discretized using second-order tetrahedral elements (C3D10M), while ligaments were modeled using triangular and quadrilateral shell elements (S3R and S4R). Shell element thicknesses were assigned based on anatomical data. To ensure simulation accuracy and numerical convergence, element size and mesh quality were carefully controlled. The maximum element size for both solid and shell elements was set to 2 mm.

**Figure 1.**
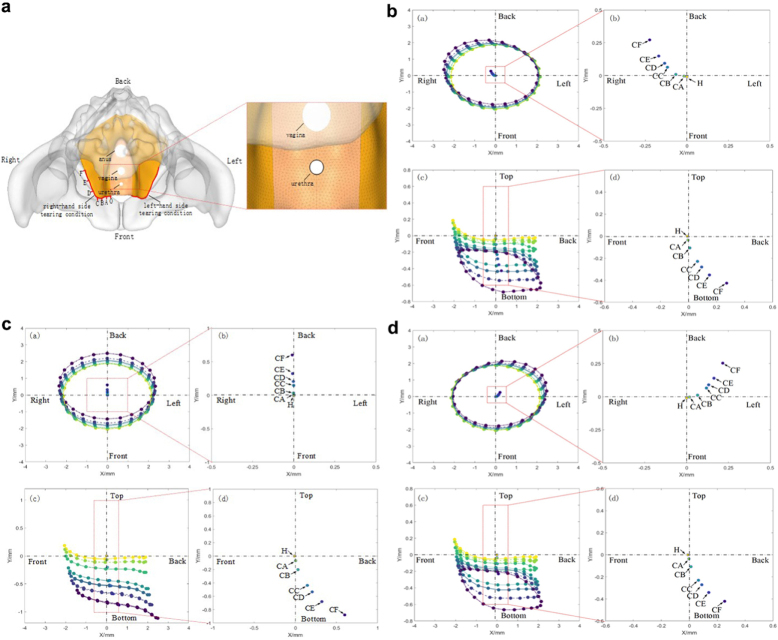
Displacement of the urethra with different conditions of levator ani avulsion. (a). Diagram of levator ani muscle avulsion conditions and simulation of urethral and vagina (assumed to be circular in shape). (b–d) Urethral displacement in the right-/bilateral/LEFT- levator ani avulsion. For each figure of b–d, upper left, top view from head to toe; upper right, partial enlargement of the upper left one. Bottom left, side view from left to right. Bottom right, partial enlargement of the bottom left one to show the downward shift of the urethra centroid.

Three conditions were simulated to model LAA: left unilateral avulsion, right unilateral avulsion, and bilateral avulsion from the pubic rami. For each condition, six avulsion lengths were designed to represent a range of clinically plausible scenarios (Supplemental Digital Content Figure S1a, available at: http://links.lww.com/JS9/F325). To evaluate urethral displacement associated with LAA, displacement data of periurethral anatomical landmarks under varying avulsion lengths and locations were extracted. The urethra, vagina, and anus were modeled with a rounded and open configuration (Fig. 1a).

### Part II: establishment of female rodent models by simulating birth trauma

A total of 42 female virgin Sprague–Dawley (SD) rats, aged 8 weeks and with a mean body weight of 249.2 g, were used in this study. The experimental procedures were reported in accordance with the ARRIVE guidelines (Animals in Research: Reporting *In Vivo* Experiments)^[8]^. All animals underwent balloon inflation to simulate birth trauma. Prior to the procedure, the bladder was emptied using a 22G transurethral Intracath catheter (Becton Dickinson, Sandy, Utah). A modified 22Fr Foley catheter was then inserted intravaginally and inflated with 5 mL of distilled water. A 100 g weight was attached to the distal end of the catheter. The rat was placed in a prone position with the pubic symphysis aligned at the edge of the table for 3 hours. The symphysis served as a fulcrum, allowing the catheter and weight to hang freely without contacting the table surface (Supplemental Digital Content Figure S2, available at: http://links.lww.com/JS9/F325).

Fourteen days after balloon inflation, all rats underwent sneeze testing and transurethral urodynamic evaluation, following the methodology established by Sievert *et al*^[9]^. At least three measurements per animal were obtained, and the mean value was used for data analysis.

**HIGHLIGHTS**

- This study proposes the urethral deviation theory as a novel mechanistic explanation for stress urinary incontinence (SUI), thereby complementing the classical hammock theory.
- The classical hammock theory posits that urethral hypermobility contributes to the development of SUI, which can be interpreted as a form of vertical urethral deviation. Our findings further demonstrate that unilateral levator ani avulsion may result in lateral urethral displacement during episodes of increased abdominal pressure, suggesting that horizontal urethral deviation may also represent a relevant pathophysiological mechanism in SUI.
- Preventing levator ani avulsion during childbirth is crucial. Women diagnosed with levator ani injuries should undergo appropriate rehabilitation training and implement lifestyle modifications to reduce the risk of developing SUI.

Immediately after euthanasia, the pubococcygeus muscle was harvested. Following gas anesthesia, the muscle was carefully dissected and prepared for histological and ultrastructural examination. Hematoxylin and eosin (HE) staining and electron microscopy were performed on pubococcygeus muscle samples from each experimental group.

### Part III: clinical survey on female SUI patients

This was a dual-tertiary center, cross-sectional study involving 4125 women who presented to the clinic with lower urinary tract symptoms between January 1, 2016, and December 31, 2021. The study was reported in accordance with the STROCSS criteria^[10]^.

The study population consisted of volunteer participants who met the inclusion criteria. The inclusion criteria were as follows: (1) presence of lower urinary tract symptoms only; (2) completion of urodynamic studies and transperineal ultrasound during the visit; (3) history of vaginal delivery with no use of forceps or vacuum extraction during delivery; (4) availability of complete data; and (5) absence of other systemic diseases.

Women were excluded if they met any of the following criteria: (1) diagnosis of mixed urinary incontinence or bladder dysfunction; (2) recurrent urinary tract infections; (3) prior surgical treatment for SUI, complex urethral surgery, or pelvic surgery; (4) neurologic disorders, poorly controlled diabetes, or senile dementia; (5) known pelvic organ prolapse; (6) urogenital fistula or urethrocele; (7) post-void residual urine volume ≥100 mL; or (8) incomplete data collection.

All observers were blinded to patient group assignments, particularly for subjective measurements.

Transperineal ultrasound was performed using a Voluson E8/E10 Expert system (GE Healthcare Ultrasound, Milwaukee, WI, USA) with a RAB6-D/RM6C transducer, under the supervision of three senior sonographers. Operators were blinded to all clinical and previous ultrasound data. Pelvic floor ultrasound images were acquired following previously established protocols^[6]^, in accordance with the standard technique described by Hans Peter Dietz^[11]^. LAA was diagnosed using the method described by Dietz *et al*^[12]^. The levator-urethral gap (LUG), defined as the distance between the urethra and the levator ani muscle at its insertion on the pubic rami, was measured. LAA was defined as an LUG> 25 mm across all three central slices.

Standardized urodynamic testing was performed on all participants, including noninvasive uroflowmetry, filling cystometry in the standing position, and pressure-flow studies (PFS). Urethral pressure profilometry and Valsalva leak point pressure were also assessed. If the patient reached bladder capacity without leakage during the Valsalva maneuver, a cough test was conducted. Maximal urinary flow rate (Qmax) and detrusor pressure at Qmax (Pdet@Qmax) were recorded from the PFS.

### Statistics

As a cross-sectional study, the sample size was calculated according to the formula as follows:

$$n=(\frac{Z_{1-\alpha /2}*\;\sigma }{\delta })$$

where *n* denotes the sample size, δ denotes the admissible error, and σ denotes the variation index.

According to the literature^[2–4]^, the expected population rate was 0.1057. We estimated σ = 5, δ = 0.03, α = 0.05, and then the sample size was at least 404. In consideration of 10% of lost to follow-up, the sample size was estimated to be 449.

Statistical analysis was conducted after assessing data normality using the Kolmogorov–Smirnov and Shapiro–Wilk tests. One-way analysis of variance (ANOVA) was applied to compare normally distributed continuous variables, while the Kruskal–Wallis test was used for variables with non-normal distributions. Contingency tables combined with the Chi-squared test were employed to evaluate associations between categorical variables. Multiple comparisons were adjusted using the Bonferroni correction method. Odds ratio (OR) analysis was also performed to assess the strength of the association between LAA and SUI. All statistical analyses were carried out using SPSS 20.0 software for Windows.

### Ethics approval and consent to participate

All animal protocols were approved by the Animal Care and Use Committee of () (Registration Number: ()). The clinical data used in this study were obtained in accordance with the regulations of the National Health Commission (Registration Number: ()) and registered with the () Clinical Trial Registry (Registration Number: ()). All participating patients provided written informed consent prior to enrollment. Additionally, written consent was obtained from all patients for the publication of their case details, including the use of identifiable images.

## Results

### Part I: female pelvic FEM revealed ipsilateral urethral displacement toward the avulsed direction in unilateral levator ani avulsion

A female pelvic FEM was established. Figure 1 presents the results of the finite element analysis, illustrating the displacement patterns of the levator ani muscle under various avulsion conditions. Under simulated intra-abdominal pressure (1 kPa), the displacement of the ipsilateral levator ani muscle increased proportionally with the severity of unilateral LAA, while the contralateral side showed minimal change (Supplemental Digital Content Figure S1 b & d, available at: http://links.lww.com/JS9/F325). In cases of bilateral levator ani avulsions, the displacement became more symmetric (Supplemental Digital Content Figure S1c, available at: http://links.lww.com/JS9/F325).

Urethral displacement under different avulsion conditions is also shown in Figure 1. The analysis revealed that in unilateral LAA, the urethral centroid shifted significantly toward the direction of the avulsion (downward and backward), with the degree of displacement increasing with the length of the tear. The entire urethra exhibited elongation, with its lateral border elevated relative to the medial border in a vertical orientation (Figure 1b and d). In contrast to unilateral LAA, bilateral avulsion resulted in nearly symmetrical urethral deformation without torsion, primarily involving posterior and inferior displacement with minimal lateral deviation (Fig. 1c).

### Part II: rat models simulated with birth trauma demonstrated that rats with LAA are more susceptible to developing SUI

Under identical experimental conditions, 66.67% (28/42) of rats were confirmed to have levator ani injuries based on hematoxylin and eosin staining and electron microscopy results (Supplemental Digital Content Figure S3, available at: http://links.lww.com/JS9/F325), including 17 with unilateral avulsion and 11 with bilateral avulsion. Based on these findings, the rats were divided into a study group (with LAA, *n* = 28) and a control group (without LAA, *n* = 14).

In the study group, 18 rats exhibited a positive cough test result, including 13 with unilateral avulsion and 5 with bilateral avulsion. In contrast, only four rats in the control group showed a positive cough test. These results indicate that rats with LAA are significantly more likely to develop SUI following simulated birth trauma compared to those without LAA (*P* = 0.031), particularly in cases of unilateral LAA (*P* = 0.011).

Urodynamic analysis revealed that the study group had significantly lower internal urethral pressure (IUP), abdominal leak point pressure, and intravesical pressure (IVP) compared to the control group (*P* < 0.001, *P* = 0.014, and *P* = 0.009, respectively). However, no significant difference was observed in maximum bladder capacity between the two groups (*P* = 0.431) (Supplemental Digital Content Figure S4, available at: http://links.lww.com/JS9/F325).

### Part III: risk factors for SUI in women

**Figure 2.**
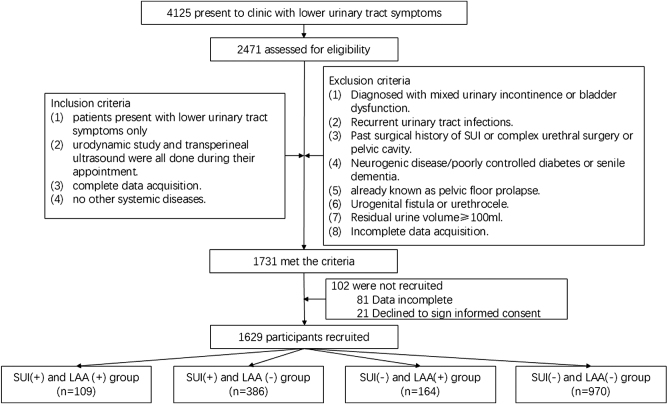
The process of capturing patients.

**Figure 3.**
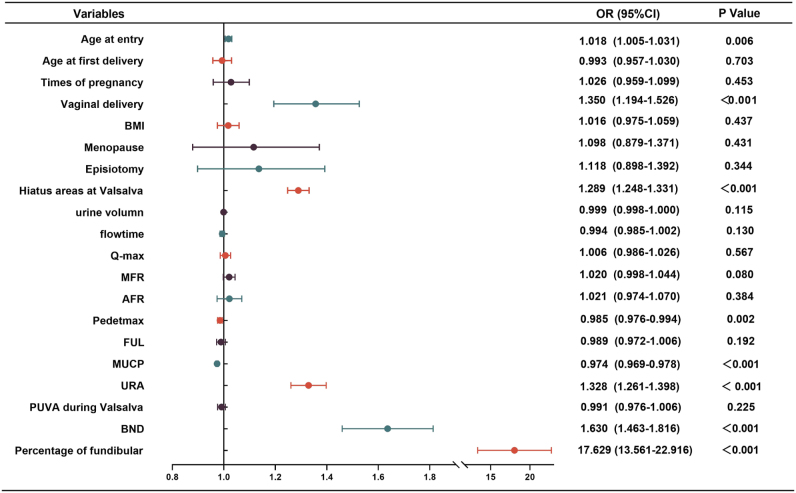
Forest map shows all potential risk factors of SUI. OR, odds ratio; BMI, body mass index; Q-max & MFR, maximal flow rate; AFR, average flow rate; FUL, functional urethral length; MUCP, maximum urethral closure pressure; URA, urethral rotation angle; PUVA, posterior vesicourethral angle; BND, bladder neck descent.

**Figure 4.**
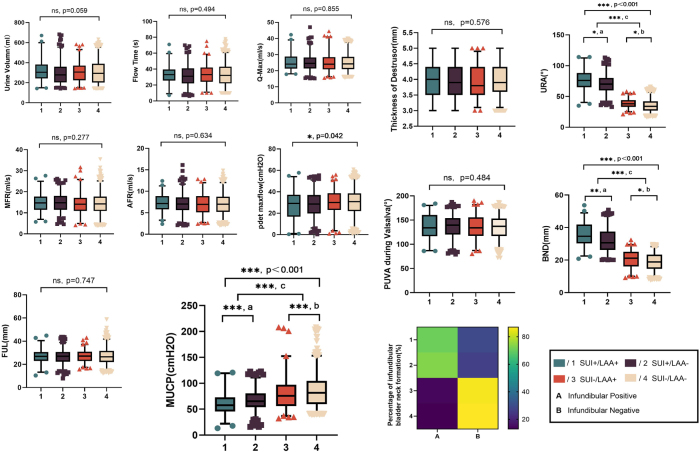
All measurements of pelvic floor ultrasound and urodynamics were compared with different groups. MFR, maximal flow rate; AFR, average flow rate; FUL, functional urethral length; MUCP, maximum urethral closure pressure; URA, urethral rotate angle; PUVA, posterior vesicourethral angle; BND, bladder neck descent.

A total of 1629 participants were ultimately recruited (Fig. 2) and categorized into four groups. The general clinical characteristics of the patients are summarized in Table 1. Figure 3 presents the OR for all identified risk factors associated with SUI. Advanced maternal age and a higher number of vaginal deliveries appeared to be associated with an increased likelihood of developing SUI. Furthermore, women with both SUI and LAA exhibited higher values of urethral rotation angle and bladder neck descent on transperineal ultrasound, and lower maximum urethral closure pressure on urodynamic testing, compared to those with isolated SUI (Fig. 4).

“a” represented the comparison between two SUI+ groups, namely, the comparison between the SUI+/LAA+ group and the SUI+/LAA− group; “b” denoted the comparison between two SUI− groups, that is, the comparison between the SUI−/LAA+ group and the SUI−/LAA− group; “c” indicated the comparison between the SUI+ group and the SUI− group.

**Table 1:** General conditions of patients involved in this study

Characteristic	Stress urinary incontinence present		Stress urinary incontinence absent		*P* value
	Levator avulsion (+) *n* = 109	Levator avulsion (-) n = 386	Levator avulsion (+) n = 164	Levator avulsion (-) n = 970	
Age at entry, y					0.440
<40	19 (17.4)	64 (16.6)	15 (9.1)	118 (12.2)	
40–<50	30 (27.5)	105 (27.2)	65 (39.6)	319 (32.9)	
50–<60	31 (28.4)	120 (31.1)	64 (39.0)	425 (43.8)	
≥60	29 (26.6)	97 (25.1)	20 (12.2)	108 (11.1)	
Age at first delivery, y					0.973
<30	100 (91.7)	354 (91.7)	151 (92.1)	874 (90.1)	
30–<35	8 (7.4)	26 (6.7)	11 (6.7)	89 (9.2)	
≥35	1 (0.9)	6 (1.6)	2 (1.2)	7 (0.7)	
Vaginal delivery					<0.001
1	39 (35.8)	191 (49.5)	77 (47.0)	597 (61.6)	
2	34 (31.2)	90 (23.3)	45 (27.4)	227 (23.4)	
≥3	36 (33.0)	105 (27.2)	42 (25.6)	146 (15.0)	
BMI, kg/m^2^					0.398
<18.5	3 (2.8)	12 (3.1)	4 (2.4)	21 (2.2)	
18.5–<24	66 (60.5)	242 (63.7)	111 (67.7)	649 (66.9)	
24–<28	35 (32.1)	113 (29.3)	44 (26.8)	260 (26.8)	
≥28	5 (4.6)	19 (4.9)	5 (3.0)	40 (4.1)	
Menopause	73 (67.0)	254 (65.8)	106 (64.6)	619 (63.8)	0.856
Episiotomy	79 (72.5)	239 (61.9)	115 (70.1)	584 (60.2)	0.012
Hiatus areas at Valsalva, cm^2^					<0.001
<20	6 (5.5)	53 (13.7)	10 (6.1)	426 (43.9)	
20–<25	31 (28.5)	148 (38.4)	65 (39.6)	467 (48.2)	
25–<30	47 (43.1)	127 (32.9)	74 (45.1)	76 (7.8)	
≥30	25 (22.9)	58 (15.0)	15 (9.2)	1 (0.1)	
IIQ-7 scores^a^	42.1 (19.5)	41.6 (20.7)	29.6 (15.2)	30.3 (14.2)	<0.001
PISQ-12 scores^b^	27.5 (4.6)	28.0 (3.2)	28.2 (3.3)	28.8 (3.3)	0.148

BMI, body mass index; IIQ, Incontinence Impact Questionnaire; PISQ, Patient-Initiated Sexual Questionnaire.

^a^ Sample size is 81 for stress urinary incontinence present/levator avulsion (+) group, 255 for stress urinary incontinence present/levator avulsion(−) group, 107 for stress urinary incontinence absent/levator avulsion(+) group, 732 for stress urinary incontinence absent/levator avulsion(**−**) group.

^b^ Sample size is 54 for stress urinary incontinence present/levator avulsion(+) group, 129 for stress urinary incontinence present/levator avulsion(**−**) group, 79 for stress urinary incontinence absent/levator avulsion(+) group, 521 for stress urinary incontinence absent/levator avulsion(**−**) group.

Table 2 outlines the distribution of SUI-positive (SUI+) and SUI-negative (SUI−) groups, along with the corresponding OR for different types of LAA in relation to SUI. The data indicate a higher prevalence of LAA in the SUI+ group compared to the SUI− group. Consistent with previous findings^[6,7]^, unilateral avulsion – regardless of side – emerges as a significant risk factor for SUI. In contrast, bilateral avulsion does not demonstrate a statistically significant association with SUI.

**Table 2:** The proportions of LAA in SUI group and the odds ratios for SUI

	Proportion in SUI+ group^a^, *n* (%)	Proportion in SUI− group^b^, *n* (%)	Odds ratios for SUI		
			Odds ratios	95% CI	*P*-value
Uni-avulsion					
L-avulsion	52 (10.51%)	68 (6.00%)	1.882	1.290–2.747	0.001266
R-avulsion	42 (8.48%)	62 (5.47%)	1.639	1.091–2.463	0.019887
Uni-avulsion in total	94 (18.99%)	130 (11.46%)	1.857	1.389–2.481	0.00004
Bilateral avulsion	15 (3.03%)	34 (3.00%)	1.033	0.557–1.914	0.876
Avulsion in total	109 (22.02%)	164 (14.46%)	1.715	1.309–2.246	0.00012

^a^ SUI+ group, SUI positive group represents women diagnosed with SUI, in the present study, it means SUI+/LAA+ group and SUI+/LAA**−** group (*n* = 495).

^b^ SUI− group, SUI negative group represents women confirmed without suffering from SUI, in this study, it means SUI**−**/LAA+ group and SUI**−**/LAA− group (*n* = 1134).

SUI, stress urinary incontinence; CI, confidence intervals; L, left; R, right.

To further explore potential etiological mechanisms linking unilateral LAA to SUI, we analyzed the LUG measurements.

**Figure 5.**
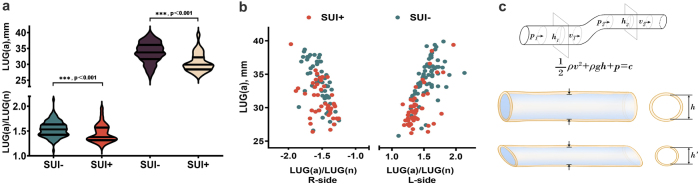
Urethral deviation hypothesis explained by LUG measurements and Bernoulli formula. (a, b) LUG measurements and ratios of LUG (avulsion-side)/LUG (normal side). (LUG(A)/LUG(N)) were compared between SUI-positive (SUI+) and SUI-negative (SUI−) group. (c) Diagrammatic drawing of urethral deviation hypothesis explained by Bernoulli formula.

The results revealed that a smaller LUG value in unilateral LAA (left or right), as well as a lower ratio of LUG(A) to LUG(N) – indicating a closer anatomical proximity of the avulsion edge to the urethral opening – is significantly correlated with an increased risk of SUI (see Fig. 5a and b). This observation aligns with the findings from our previous finite element analysis.

## Discussion

In this study, we propose the hypothesis of urethral deviation as a potential contributing mechanism in the pathogenesis of SUI. A combination of female pelvic FEM, rodent animal models, and a large clinical population was utilized to investigate the underlying mechanisms of female SUI. The findings from all three components consistently demonstrate that unilateral LAA is a significant risk factor for SUI. Furthermore, urethral deviation induced by unilateral LAA may represent an additional etiological mechanism of SUI, complementing the classical fascial hammock theory.

### LAA may play an important role in the increased prevalence of SUI following vaginal childbirth

As reported, risk factors for SUI include age, pregnancy, vaginal delivery, and higher BMI^[2,13,14]^. In the present study, both age and vaginal delivery were identified as significant risk factors for SUI, whereas pregnancy and BMI did not show statistically significant associations among the studied population. The lack of significance for pregnancy may be attributed to the presence of early induced abortions and minimal physiological impact during gestation. Additionally, although the majority of women included in this study had BMIs within the normal range (18.5–24), as defined by the World Health Organization, 28% of the participants had a BMI exceeding 25 (overweight). Nevertheless, our results did not reveal a significant association between BMI and SUI.

Vaginal childbirth is a well-established contributor to the development of SUI^[15,16]^. It is known that LAA occurs in 15%–30% of women following vaginal delivery^[17,18]^. However, the relationship between LAA and SUI remains controversial. A systematic review including studies published before November 5, 2019, concluded that there is no clear association between LAA and SUI in women, while also acknowledging the limited number of studies in this area^[19]^. In contrast, Frisch *et al*^[20]^ suggested that widening of the levator hiatus after childbirth may contribute to urinary incontinence. Chan *et al*^[21]^ did not find any association between LAA and SUI during the first year postpartum. However, in a follow-up study conducted three years later, they reported that women with LAA were significantly more likely to be diagnosed with SUI within 3–5 years postpartum^[22]^. Aly Youssef^[23]^ also reported that LAA appears to be associated with an increased risk of postpartum SUI. Yan’s research^[24]^, based on a 1:1 case-control study, demonstrated that pelvic floor muscle injury and dysfunction are more likely to lead to hypermobility of the bladder neck and urethra, thereby contributing to the development of SUI. Wu *et al*^[25]^ further showed that pregnancy-induced connective tissue laxity and avulsion of the pubococcygeus muscle are more likely to trigger SUI.

As previously demonstrated, our earlier research indicated that LAA may serve as a risk factor for SUI^[6]^. In the present study, women with LAA were 1.715 times more likely to be diagnosed with SUI compared to those without LAA. Therefore, we conclude that LAA, which commonly occurs after vaginal delivery, is associated with the prevalence of SUI. Patients with LAA are more likely to develop SUI (OR = 1.715, 95% confidence interval: [1.309–2.246], *P* < 0.001). When unilateral and bilateral avulsions were analyzed separately, an interesting finding emerged: unilateral avulsion was significantly associated with SUI, whereas bilateral avulsion was not – a result consistent with our previous findings^[6]^.

### Hypothesis of LAA in the development of SUI

The urethra is supported by a layer composed of the endopelvic fascia and anterior vaginal wall, which provides hammock-like structural support. This layer gains mechanical stability through its lateral attachment to the arcus tendineus fasciae pelvis and the levator ani muscle^[26]^. Several studies have explored potential etiological mechanisms linking LAA to SUI. It has been reported that women with levator ani injury exhibit 24% lower urethral closure pressure during maximal pelvic floor contraction compared to those without such injury^[27]^, a finding consistent with our urodynamic results. According to the “hammock hypothesis,” injury to one pelvic floor structure may predispose adjacent structures to damage or dysfunction^[28–30]^. While reduced urethral closure function is observed in some women after LAA, it is not universal – suggesting a “field effect” in which injury in one area (e.g. LAA) increases the likelihood of injury or dysfunction in adjacent structures (e.g. the urethra)^[9]^.

In the present study, we propose that patients with unilateral LAA, rather than bilateral LAA, are more susceptible to SUI due to alterations in urethral support leading to urethral deviation. This phenomenon was corroborated by our FEM analysis. Our findings suggest that unilateral injury to the levator ani muscle may explain the increased vulnerability to SUI, particularly when the avulsion edge’s attachment point is in closer proximity to the urethral opening, which correlates with a higher likelihood of stress incontinence. This observation aligns with our previous investigations^[6]^, which indicated that disruption of the levator ani muscle may compromise stress continence due to the loss of additional occlusive force on the urethral wall, especially when the injury is asymmetric and leads to structural instability. In contrast, bilateral avulsion may result in a more balanced anatomical configuration, thereby exerting less influence on intra-abdominal pressure transmission.

Delancey *et al*^[31]^ have observed that in patients with urinary incontinence, the pubococcygeus muscle loses its symmetric morphology, showing lateral displacement, thinning, or even complete atrophy. Such impairment may contribute to SUI, as the urethral wall experiences diminished compressive force, particularly during episodes of increased intra-abdominal pressure^[32]^. Furthermore, based on MRI analyses, Prando^[33]^ and Kim^[34]^ independently reported asymmetry, lateral displacement, and thinning or attenuation of the pubococcygeus muscle in patients with SUI. These morphological changes are indirectly consistent with our urethral deviation hypothesis, as the muscle on the affected side becomes thinner and undergoes posterior-inferior displacement.

Building upon these findings, we propose the hypothesis of urethral deviation as a contributing mechanism in the pathogenesis of SUI. According to the fundamental principles of SUI, IUP is lower than IVP. During sudden increases in abdominal pressure (e.g. coughing, Valsalva maneuver), urine may be involuntarily expelled from the bladder. Our hypothesis can be further explained using the Bernoulli principle. In the presence of unilateral LAA, an unstable pelvic support structure may lead to uneven pressure distribution during increased intra-abdominal pressure, resulting in urethral deviation. As illustrated in Figure 5c, according to the Bernoulli principle, a reduction in cross-sectional area leads to an increase in flow velocity and a corresponding decrease in pressure. In accordance with our urethral inclination theory, varying degrees of unilateral levator ani muscle tear may cause imbalanced intra-abdominal pressure between the two sides, resulting in posterior and inferior displacement of the urethra. However, the anatomical origin and insertion points of the urethra remain fixed. Consequently, the overall inclination of the urethra leads to its elongation, and the cross-sectional area becomes smaller compared to the non-displaced state (the middle panel of Figure 5c represents the normal condition, while the lower panel shows urethral inclination). A smaller cross-sectional area implies a reduction in the relative lumen height (h). According to the formula, with c as a constant, when height (h) decreases, velocity (v) increases. As velocity increases, the urinary flow rate per unit time rises, indicating a decrease in urethral resistance during micturition.

### Clinical implications

The urethral deviation theory effectively complements the limitations of the hammock theory, offering additional insights into the pathogenesis of female SUI. In patients who do not exhibit anterior vaginal wall prolapse or visible urethral descent but still experience persistent SUI, the urethral deviation hypothesis suggests that SUI may arise from urethral tilt caused by unilateral avulsion of the levator ani muscle, even in the absence of significant urethral descent.

Following pelvic floor reconstruction surgery, although high urethral mobility may be reduced, new-onset SUI can still occur. The urethral tilt induced by surgical intervention may be one of the contributing factors to postoperative SUI development.

Currently, midurethral sling placement remains the gold standard for the surgical management of SUI. However, the introduction of the urethral deviation theory provides novel perspectives on the role of autologous tissue repair – particularly the repair of levator ani muscle injuries – in SUI surgery. This approach may help reduce reliance on polypropylene slings and lower the risk of associated complications. For instance, could the levator ani muscle repair emerge as a viable alternative or adjunctive treatment for SUI? Is it feasible to perform levator ani muscle repair concurrently with surgeries for pelvic organ prolapse, vaginal tightening, or other vaginal procedures? Moreover, is TVT surgery considered the gold standard for SUI because, compared with other surgical options, TVT not only prevents downward displacement of the urethra but also concurrently addresses its lateral deviation? These questions represent important areas for further investigation in future studies.

This study has several limitations. First, as a two-center study, it may not fully represent the broader patient population. Second, further investigations using fluid dynamics simulations are required to validate the hypothesis of urethral deviation.

## Conclusions

In summary, urethral deviation associated with LAA may provide an additional mechanistic perspective to the hammock theory of SUI, particularly in the context of unilateral LAA. This insight helps explain specific clinical scenarios that are not fully accounted for by the hammock theory, such as the occurrence of SUI in the absence of pelvic organ prolapse and the development of *de novo* SUI following pelvic floor reconstruction surgery.

Given that LAA has been identified as a risk factor for female SUI, these findings suggest the importance of minimizing the risk of LAA during childbirth. Women diagnosed with levator ani injuries should be encouraged to undergo pelvic floor rehabilitation training and adopt appropriate lifestyle modifications to reduce the likelihood of developing SUI.

## Data Availability

The data tha support the findings of this study are not publicly available due to privacy and ethical restrictions. They may only be available on request from the corresponding author. A data request and brief analysis plan will be required in accordance with the ethics committee requirements. These will be reviewed by the lead, study steering committee, and study sponsor. A data transfer agreement will have to be completed before any data is shared. After completion of the data transfer agreement, data will be shared as password-protected files. Data sharing will abide by the rules and policies defined by the sponsor, relevant institutional review boards, as well as local, state, and federal laws and regulations. The rights and privacy of individuals participating in the research will be protected at all times.

## Research registration unique identifying number (UIN)

For the studies using all the clinical data were approved by the National Health Commission (MR-50-23 037229), the Chinese clinical trial registry (ChiCTR2400095028).

## Presentation

Not commissioned, externally peer-reviewed.
